# Uncoupled responses of Smad4-deficient cancer cells to TNFα result in secretion of monomeric laminin-γ2

**DOI:** 10.1186/1476-4598-9-65

**Published:** 2010-03-22

**Authors:** Dirk Zboralski, Bettina Warscheid, Susanne Klein-Scory, M Bassel Malas, Heiko Becker, Miriam Böckmann, Helmut E Meyer, Wolff Schmiegel, Patricia Simon-Assmann, Irmgard Schwarte-Waldhoff

**Affiliations:** 1 Medizinische Universitätsklinik, Knappschaftskrankenhaus, IMBL, Ruhr-Universität Bochum, Bochum, Germany; 2 Medizinisches Proteom-Center, Ruhr-Universität Bochum, Bochum, Germany; 3 Abtlg. Gastroenterologie und Hepatologie, Kliniken Bergmannsheil, Ruhr-Universität Bochum, Bochum, Germany; 4 Inserm, U682, Strasbourg, F-67200 France; Univ Strasbourg, UMR-S682, Strasbourg, F-67081 France

## Abstract

**Background:**

Functional loss of the tumor suppressor Smad4 is involved in pancreatic and colorectal carcinogenesis and has been associated with the acquisition of invasiveness. We have previously demonstrated that the heterotrimeric basement membrane protein laminin-332 is a Smad4 target. Namely, Smad4 functions as a positive transcriptional regulator of all three genes encoding laminin-332; its loss is thus implicated in the reduced or discontinuous deposition of the heterotrimeric basement membrane molecule as evident in carcinomas. Uncoupled expression of laminin genes, on the other hand, namely overexpression of the laminin-γ2 chain is an impressive marker at invasive edges of carcinomas where tumor cells are maximally exposed to signals from stromal cell types like macrophages. As Smad4 is characterized as an integrator of multiple extracellular stimuli in a strongly contextual manner, we asked if loss of Smad4 may also be involved in uncoupled expression of laminin genes in response to altered environmental stimuli. Here, we address Smad4 dependent effects of the prominent inflammatory cytokine TNFα on tumor cells.

**Results:**

Smad4-reconstituted colon carcinoma cells like adenoma cells respond to TNFα with an increased expression of all three chains encoding laminin-332; coincubation with TGFβ and TNFα leads to synergistic induction and to the secretion of large amounts of the heterotrimer. In contrast, in Smad4-deficient cells TNFα can induce expression of the γ2 and β3 but not the α3 chain. Surprisingly, this uncoupled induction of laminin-332 chains in Smad4-negative cells rather than causing intracellular accumulation is followed by the release of γ2 into the medium, either in a monomeric form or in complexes with as yet unknown proteins. Soluble γ2 is associated with increased cell migration.

**Conclusions:**

Loss of Smad4 may lead to uncoupled induction of laminin-γ2 in response to TNFα and may therefore represent one of the mechanisms which underlie accumulation of laminin-γ2 at the invasive margin of a tumor. The finding, that γ2 is secreted from tumor cells in significant amounts and is associated with increased cell migration may pave the way for further investigation to better understand its functional relevance for tumor progression.

## Background

In normal tissues, the epithelium is separated from the underlying mesenchyme by the basement membrane (BM), a specialized sheet of the extracellular matrix. The BM is built from constituents produced by both the epithelial and the mesenchymal cells [1,2]. Whereas collagen IV is the most prominent mesenchymal derived component providing the structural scaffold of the BM sheet the epithelial derived laminins build the centerpiece of the network that harbors additional proteins including perlecan, nidogen and fibulin [3]. The basement membrane has been recognized as a structural but also as an important functional component of tissues. In particular, the laminins mediate cellular functions including adhesion, migration, growth and tissue-specific gene expression [4,5].

The laminins are large heterotrimeric glycoproteins with at least 15 different isoforms composed of different combinations of one α-, one β- and one γ-chain, each, out of five α, three β and three γ-chains. The laminins are expressed in a tightly regulated development- and differentiation-specific pattern [6-8]. In the adult human intestine, laminins-211 and -511 show complementary distributions along the crypt-villus axis, whereas laminin-332 is restricted to the villus regions. In premalignant stages of colorectal carcinogenesis, namely in different types of adenomas, normal expression and deposition of laminin-332 and -511 has been reported. The transition to malignancy is defined by breaking the basement membrane barrier. In colorectal carcinomas, this is associated with a lack of laminin-511 and with irregular deposition of laminin-332 at invasive edges [9-11]. Relative overexpression of the laminin-γ2 (and β3) chain has often been described and represents one of the most impressive molecular markers for the invasive front of colorectal and other cancer entities (for review see [12]). It specifically marks socalled budding tumor cells [13,14]. Laminin-γ2 has been described as a target gene of the Wnt/β-catenin pathway [15]. Whereas β-catenin is constitutively activated through mutation of the tumor suppressor APC in the majority of adenomas the relative overexpression of γ2 at the invasive edge of carcinomas requires additional alterations. Overexpression of γ2 is believed to result from cellular responses to environmental signals illustrating that the regulation of laminin expression is subject to tumor cell intrinsic factors including the pattern of their respective genetic alterations and to extrinsic microenvironmental factors including signals from inflammatory cells in the tumor tissue.

We have recently identified laminin-332 as a target structure of the tumor suppressor Smad4 [16]. We have shown that Smad4 functions as a positive transcriptional regulator of all three chains encoding laminin-332. Reexpression of Smad4 led to the increased expression of heterotrimeric laminin-332 and to its deposition in basement membrane-like structures at contact sites with fibroblasts. Loss of Smad4 in the carcinogenic process, in turn, is implicated in reduced or absent expression of laminin-332 in poorly differentiated carcinomas.

Smads are primarily characterized as transmitters of signals from the TGFβ superfamily of cytokines but also function as promiscuitive transcriptional coregulators that can interact with a variety of ubiquitous and tissue-specific transcription factors and coregulators in a context-dependent manner [17,18]. TGFβ, in Smad4-reexpressing cancer cells like in premalignant adenoma cells induces the expression of all three genes encoding heterotrimeric laminin-332 whereas Smad4-negative cells are non-responsive [16,19]. The underlying molecular mechanisms are surprisingly complex and involve transcription factor binding sites like AP1 which are targeted by various signaling cascades. Moreover, the modular composition of the three promoters significantly differs from each other; a functional smad binding element (SBE) is present exclusively in the LAMA3 promoter [19]. Thus, we wonder if the consequences of Smad4 loss in response to extracellular signals other than TGFβ may differ between the three genes encoding laminin-332. As an approach towards modelling the cytokine environment in tumor tissues we here address effects of TNFα, a prominent inflammatory cytokine produced by tumor infiltrating macrophages, on laminin-332 expression in Smad4-positive and Smad4-deficient tumor cells.

We report, that Smad4-reexpressing human colorectal cancer cells like adenoma cells respond to TNFα with a moderate increase of all three chains encoding laminin-332 and with synergistic induction in response to the combination of TGFβ and TNFα. In contrast, their Smad4-deficient counterparts display uncoupled responses to TNFα: whereas the β3 chain and in particular the γ2 chain is strongly induced in Smad4-negative cells, induction of the α3 chain is Smad4-dependent and is mediated via an NF-κB site and downstream AP1 sites in the LAMA3 promoter. Of note, TNFα induction leads to the release of significant amounts of the γ2 chain in a monomeric form and in complex with (an) unknown protein(s) as shown by Western blotting under non-reducing conditions and confirmed by mass spectrometry. Ultimately, induced secretion of soluble γ2 by transient suppression of the α3 chain leads to induction of cell migration.

## Results

### Synergistic induction of laminin-332 in human adenoma cells in response to inflammatory cytokines TGFβ and TNFα

**Figure 1:**
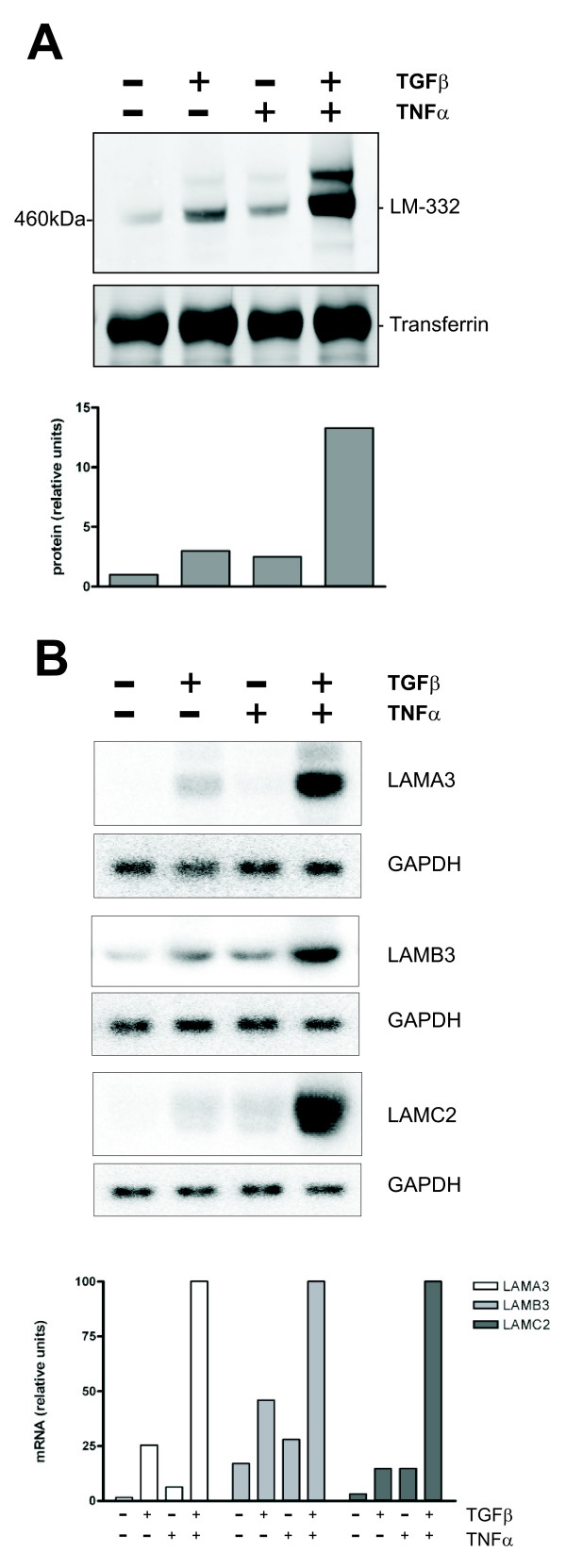
**Synergistic induction of laminin-332 in human adenoma cells in response to inflammatory cytokines TGFβ and TNFα**. **(A) **Western blot analysis of heterotrimeric laminin-332 expressed by LT97 colorectal adenoma cells. Proteins (8 μg/lane) prepared from serum-free conditioned media from LT97 cells treated with recombinant TGFβ and TNFα for 48 h as indicated were separated on 3-8% tris-acetate gradient gels (Invitrogen) under non-reducing conditions. The blot was probed with a laminin-γ2-specific antibody (polyclonal antibody 2140, PS-A) and reprobed with a transferrin-specific antibody used as a loading control. The bars indicate the relative signal strength normalized for transferrin. Note that the Odyssey detection system (LI-COR) allows for a direct digital quantification of signals. Similar results were obtained in > three experiments. The same signals, although with less sensitivity, were obtained using a commercial antibody (MAB-19562, Chemicon). **(B) **Northern blot analyses of the LAMA3, LAMB3 and LAMC2 genes prepared with RNAs from LT97 cells treated with cytokines for 24 h. Quantification of mRNA levels was done by phosphorimage analysis and signal strengths normalized with GAPDH. Similar results were obtained in three experiments.

As an approach towards modelling the microenvironment in tumor tissues we here wished to address effects of TNFα, a prominent inflammatory cytokine produced by tumor infiltrating macrophages, on laminin-332 expression of Smad4-positive and Smad4-deficient tumor cells. We use the human adenoma cell line LT97 carrying mutations of the APC and Ki-ras genes [20] as a model for early stage premalignant tumor cells with intact Smad4. We have reported previously, that LT97 cells respond to the treatment with TGFβ with transcriptional induction of all three genes encoding laminin-332 [16]. Here, LT97 cells were incubated with TNFα alone or in combination with TGFβ. There was no evidence for TNFα induced cell death in LT97 cells. Interestingly, whereas treatment with TNFα alone induced a moderate increase in the release of the laminin heterotrimer, only, the combination of both cytokines led to induced secretion of laminin-332 to an enormous extent (approximately 14-fold) (Figure 1A). Western blotting under reducing conditions with laminin chain-specific antibodies confirmed that the heterotrimer is composed of the α3, β3 and γ2 chains, as expected (data not shown). Laminin-332 specific transcripts are barely detectable in LT97 cells cultured in the absence of cytokines but are strongly induced in response to TGFβ, as reported previously [16]. TNFα treatment alone also induced the expression of all three genes. The combination of both cytokines led to a very strong synergistic induction particularly of the mRNA of α3 and γ2 chains (Figure 1B).

### Synergistic effect of TGFβ and TNFα on the secretion of heterotrimeric laminin-332 by Smad4-reconstituted human colorectal cancer cells and uncoupled responses of Smad4-deficient cells

We next sought to analyse laminin expression in Smad4-deficient and Smad4-reexpressing SW480 and SW620 human colon cancer cells in response to TNFα and to the combination of TGFβ and TNFα cytokines. SW480 cells manipulated to reexpress Smad4 after retroviral transduction have been described previously [19]; unlike SW480 cells expressing very low levels of Smad4 after stable transfection [21], moderate Smad4 overexpression in this cellular model is adequate to restore TGFβ responsiveness. Likewise, Smad4-reexpressing SW620 cell clones displayed similar restoration of TGFβ responsiveness in transient transfection assays with the currently used p3TPlux and p6SBE promoter-reporter constructs (Additional file 1).

**Figure 2:**
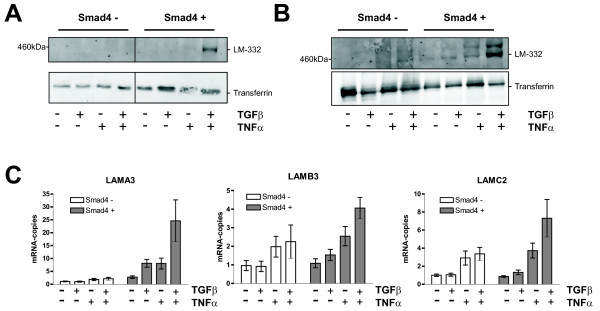
**Synergistic effect of TGFβ and TNFα on the secretion of heterotrimeric laminin-332 by Smad4-reconstituted human colorectal cancer cells and uncoupled responses of Smad4-deficient cells**. **(A and B) **Western blot analysis of proteins from conditioned media (12 μg of protein per lane) produced by SW480 (A) and SW620 (B) cells as described in figure 1. **(C) **Northern Blot analysis with RNAs from SW620 cells treated with cytokines for 4 h. Shown in each bar is the mean +/- standard error (n = 3). The additional file 2 provides additional data for SW620 cells treated with cytokines for 24 hours and data for SW480 cells treated with cytokines for 4 and 24 hours.

The secretion of laminin-332 could barely be detected in uninduced SW480 and SW620 cells and in cells induced with both cytokines separately (Figure 2A and 2B). In contrast, when cells were coinduced with both cytokines, Smad4-positive SW480 and SW620 cells but not their Smad4-negative counterparts showed secretion of significant levels of laminin-332 (Figure 2A and 2B) indicating that this response is Smad4-dependent.

We next performed expression analyses at the mRNA level in both cell lines at two different time points (at 4 and 24 h) of induction with cytokines (Figure 2C and Additional file 2). As reported previously [16,19], reexpression of Smad4 induced slight increases of basal expression levels of all three laminin-332 chains and restored their TGFβ-responsiveness (Figure 2C and Additional file 2). In contrast, TNFα induced expression of the LAMB3 and LAMC2 genes in Smad4-negative cells to a similar or even to a larger extent as compared to Smad4-positive cells. Compared to TNFα responses alone, coinduction with both cytokines did not significantly alter responses in Smad4-negative cells. In Smad4-positive cells expression of LAMB3 and LAMC2 was induced in an additive or synergistic manner by both cytokines. Of note, responses of the LAMA3 gene significantly differed from responses of the LAMB3 and LAMC2 genes: Smad4-negative cells display no or negligible induction of LAMA3 expression in response to TNFα alone and to the combination of TNFα and TGFβ in both cell lines and at both time points analysed. In contrast, Smad4-positive SW620 (but not SW480 cells) displayed responsiveness to TNFα alone (Figure 2C and Additional file 2); both, Smad4-positive SW480 and SW620 cells, showed additive or synergistic responses to the combination of both cytokines (Figure 2C and Additional file 2). This expression pattern was consistent with the strongly increased amounts of secreted laminin-332 heterotrimer by Smad4-reexpressing cells in response to combined treatment with TGFβ and TNFα. In addition, these results suggested that loss of Smad4 was responsible for uncoupled regulation of the three laminin genes in response to TNFα.

### TNFα induced secretion of monomeric laminin-γ2 by Smad4-deficient colorectal cancer cells

**Figure 3:**
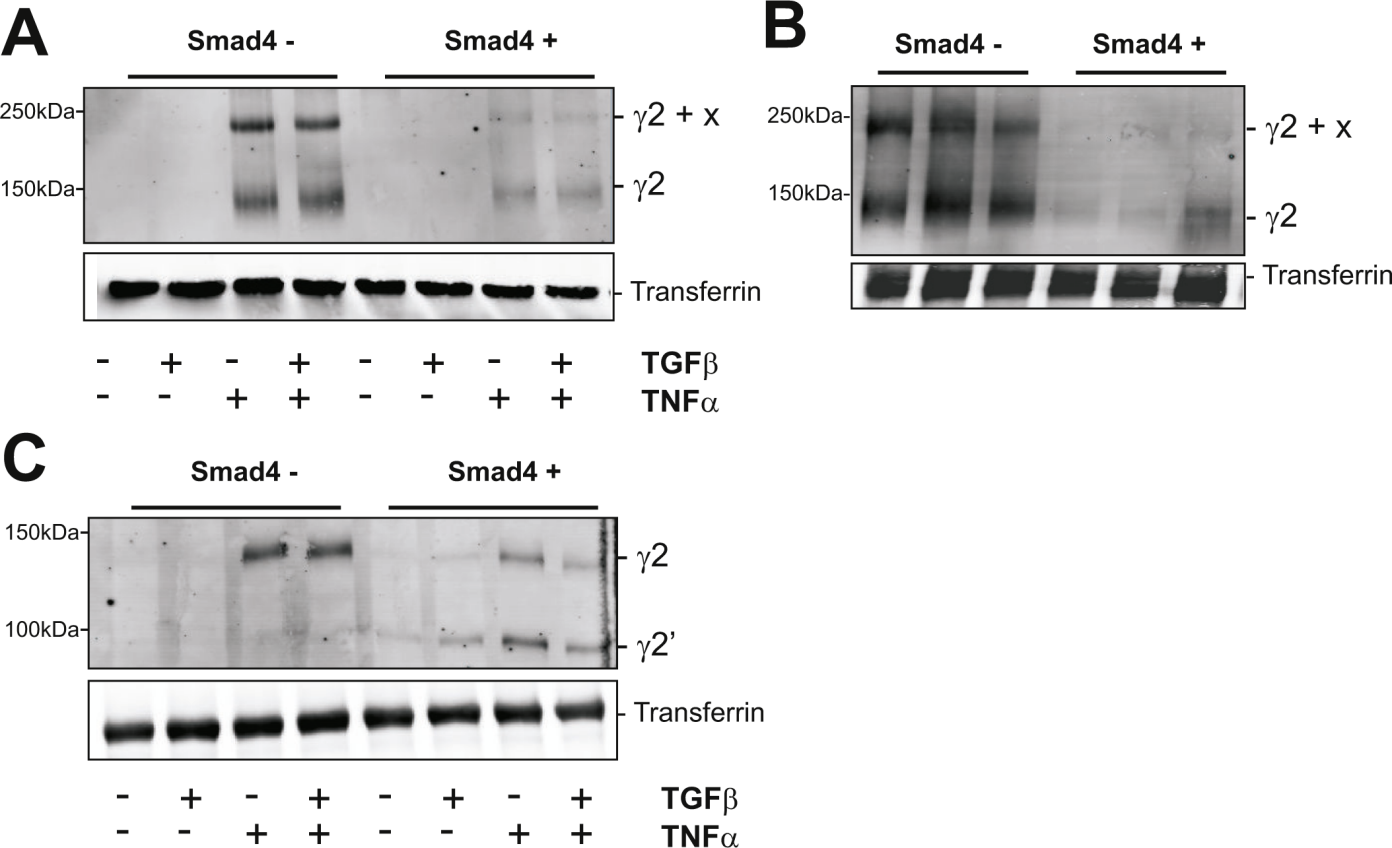
**TNFα induced secretion of monomeric laminin-γ2 by Smad4-deficient colorectal cancer cells**. **(A) **Western blot analysis of proteins from conditioned media produced by SW620 cells in response to treatment with recombinant TGFβ and TNFα for 48 h under non-reducing conditions. Proteins (16 μg/lane) were separated by SDS-PAGE on 8% polyacrylamide gels, blotted and probed with a laminin-γ2-specific antibody (2140). **(B) **A set of each, three independent Smad4-deficient and Smad4-reexpressing SW620 cell clones were treated with TNFα and conditioned media analysed like in (A). **(C) **Protein samples corresponding to those used in (A) were analyzed under reducing conditions.

Assembly of the heterotrimeric protein is believed to be a prerequisite for secretion of laminin. Having shown uncoupled induction of γ2 we searched for an intracellular accumulation of the protein by Western blotting but we could not discern specific bands in cell lysates. An analysis of the laminin-332 heterotrimer in conditioned media by Western blotting with a γ2-specific antibody under non-reducing conditions had previously shown additional diffuse signals at smaller protein sizes in lanes loaded with conditioned media proteins from TNFα treated Smad4-negative cells. A systematic analysis with gel conditions adapted revealed distinct bands corresponding to a protein size of roughly 240 and 140 kilodalton (kDa) with a commercial γ2-specific monoclonal antibody (MAB 19562, Chemicon) (data not shown). An independent γ2-specific antiserum (polyclonal, 2140) delivered an identical pattern (Figure 3A). This result was confirmed with a set of each, three independent Smad4-deficient and Smad4-reexpressing SW620 cell clones (Figure 3B). Thus, Smad4-deficient SW620 cells in response to TNFα apparently release the laminin-γ2 chain in a monomeric form and in a complex with another unknown protein. Under reducing conditions, Smad4-deficient cells showed the unprocessed form of the γ2 chain at 140 kDa, only. Laminin-γ2 chains derived from the Smad4-positive cells came as a mixture of the unprocessed form and a processed form at a size of roughly 105 kDa suggesting that processing may occur in the heterotrimeric configuration (Figure 3C).

### Mass spectrometry based confirmation of secreted laminin-γ2

**Figure 4:**
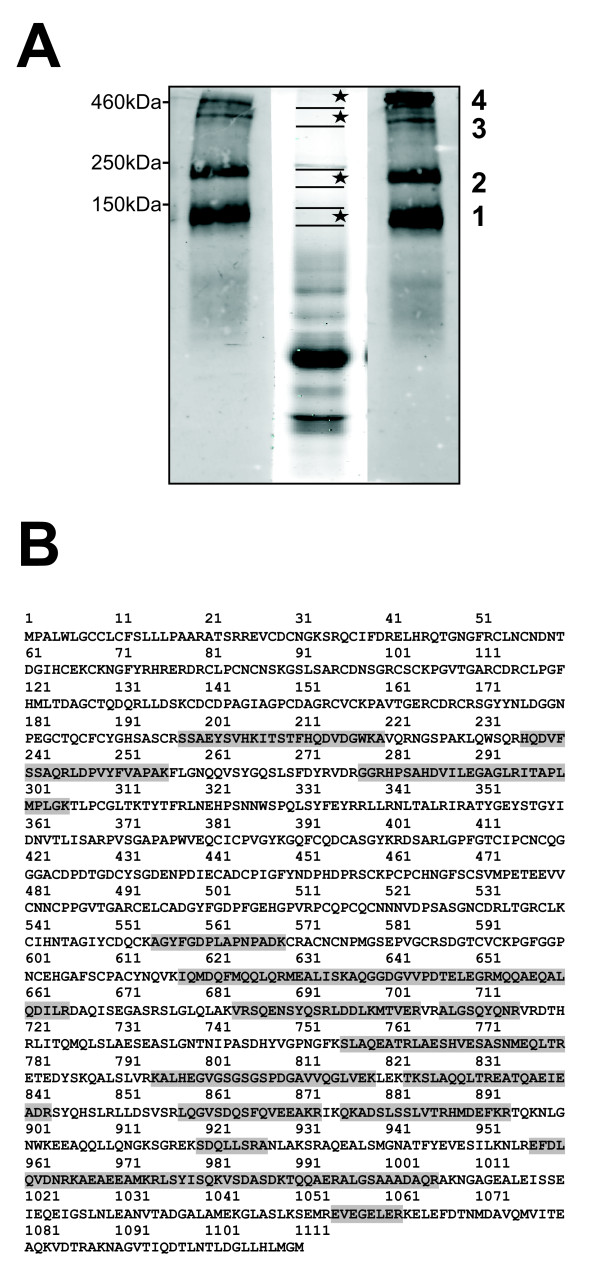
**Mass spectrometry-based confirmation of secreted laminin-γ2**. **(A) **Preparative gel electrophoresis of conditioned media from SW620 cells (Smad4-deficient) treated with TNFα. An 8% SDS-PAGE was performed with preparative amounts of protein (32 μg) in the middle lane and analytical amounts of protein (16 μg) in the left and right lanes under non-reducing conditions. The left and right lanes were used for Western blotting with a laminin-γ2-specific antibody. Four small slices (1-2 mm) corresponding to the Western blot signals were cut from the preparative gel lane and proteins analysed by mass spectrometry. **(B) **Amino acid sequence of laminin-γ2. The peptides identified by mass spectrometry in gel bands 1-4 are indicated.

To unequivocally confirm the specificity of the Western blot signals we performed proteomic analysis of conditioned media of Smad4-negative SW620 cells treated with TNFα by nanoscale liquid chromatography tandem mass spectrometry (nano-LC/MS/MS). Slices were cut form a non-reducing preparative gel corresponding to the 140 kDa signal (band 1) and to the 240 kDa region (band 2). Two slices corresponding to the putative heterotrimer signals (bands 3 and 4) were included as positive controls (Figure 4A). The laminin-γ2-specific peptides identified in gel band 1 are indicated in the amino acid sequence in figure 4B. All results of mass spectrometry and database searches are shown in additional file 3 and summarized in table 1. Among 52 proteins identified in total in gel band 1 the laminin-γ2 chain had the highest Mascot score and highest number of spectral counts which is an indirect measure for its relative abundance [22]. Likewise, laminin-γ2 ranked at position 3 according to the Mascot score among proteins identified in band 2 which corresponds to the Western blot signal at 240 kDa. Also, all three laminin chains were among the most abundant proteins in band 4 according to both their Mascot scores and spectral counts, the presumptive heterotrimer. Band number 3 which corresponds to the second slightly smaller signal and was regarded as a processed laminin-332 heterotrimer provided surprising results: Whereas the γ2 chain ranked at position 5 with 26 spectral counts, the α3 and β3 chains come at ranks 42 (spectral count 3) and 50 (spectral count 2), only, indicating that their relative amounts are much lower as compared to laminin-γ2. This, in turn, suggests that the Western blot signal at approximately 400 kDa like the signal at 240 kDa corresponds to a protein complex of laminin-γ2 with (an) as yet unknown protein(s).

**Table 1:** Mass spectrometry of proteins and protein complexes reactive with laminin-γ2 specific antibodies as indicated in Figure 4a

	band 1 140 kDa	band 2 240 kDa	band 3 400 kDa	band 4 > 460 kDa
Laminin-γ2	1* (42) [1349.7]	3* (22) [637]	5* (26) [734]	3* (40) [1305.3]
Laminin-β3	-	-	51* (2) [85.1]	4* (40) [1263.9]
Laminin-α3	38* (3) [67.1]	-	43* (3) [117]	2* (48) [1320.8]
**Total no of proteins** **identified**	52	28	82	81

*Rank, (spectral counts), [Mascot Score]; Rank according to spectral counts as a quantitative measure for the relative abundance of proteins

### Induced release of monomeric laminin-γ2 upon transient laminin-α3 knockdown in SW620 cells and its impact on cell migration

**Figure 5:**
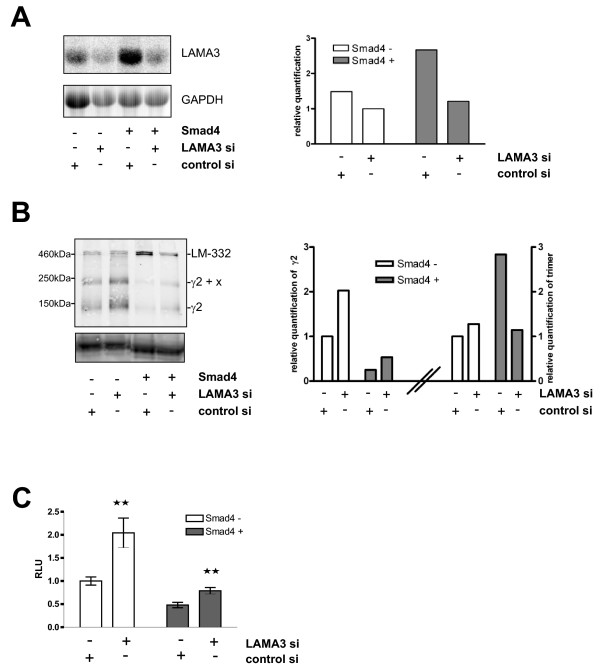
**Induced release of monomeric laminin-γ2 upon transient laminin-α3 knockdown in SW620 cells and its impact on cell migration**. **(A) **Northern blot analysis of LAMA3 knockdown in SW620 cells. RNAs were prepared from SW620 cells transiently transfected with LAMA3 siRNA and incubated with TNFα for 24 h. Quantification of LAMA3 messages normalized for GAPDH is indicated. **(B) **Western blot analysis of laminin-γ2 expression upon transient knockdown of LAMA3. SW620 cells transiently transfected with LAMA3 siRNA or non-targeting siRNA were shifted to serum-free cultures 48 h after transfection and were incubated with TNFα for another 48 h. Proteins from conditioned media (16 μg/lane) were probed with a laminin-γ2-specific antibody (2140). Quantification of the monomer and of the heterotrimer normalized for transferrin is indicated. **(C) **Migration of SW620 cells as analyzed in a transwell migration assay. SW620 cells transfected with LAMA3 siRNA or non-targeting siRNA were plated in a transwell chamber 24 h after transfection. TNFα was added one day later. Migrating cells were quantified after three days using Cell Titer Glo assay (Promega). Bars show the mean value of four experiments with the standard error of the mean. Statistical analysis was carried out by t test (one-tailed, *GraphPad Prism 4.00*).

Next, we wished to get some insight into the functional consequences of laminin-γ2 release. Accumulation of laminin-γ2 marks the invasive margin of tumors, suggesting that laminin-γ2 is associated with migratory activity. As reexpression of Smad4 induces comprehensive alterations of expression profiles and profoundly affects cellular behaviour through diverse mechanisms, the comparison of Smad4-deficient and Smad4-reexpressing cell clones is not adequate to specifically address a putative impact of monomeric laminin-γ2 on cellular migration. Therefore, we set up transient knockdown of LAMA3 expression in order to specifically induce laminin-γ2 monomer secretion in response to TNFα. In fact, transient knockdown of LAMA3 proved functional as assessed by Northern blot analysis (Figure 5A). Suppression of LAMA3 expression resulted in a twofold increase in the 240 and 140 kDa laminin-γ2 signals in conditioned media from TNFα-incubated Smad4-negative SW620 cells (and at very low levels in media from Smad4-positive cells) (Figure 5B). The heterotrimeric laminin-332 in Smad4-positive SW620 cells treated with TNFα was reduced upon transient LAMA3 knockdown as expected (Figure 5B). We then performed transwell migration assays. Both, Smad4-deficient and Smad4-positive cells displayed approximately doubled migration efficiencies upon induced release of laminin-γ2 (and reduced release of heterotrimeric laminin-332) through LAMA3 knockdown (Figure 5C).

### Smad4-dependent response of LAMA3 to TNFα is partially mediated via an NF-κB site

We have shown previously, that the molecular mechanisms underlying Smad4-mediated TGFβ responses significantly differ between the three promoters; specifically, the LAMA3 promoter, only, harbours a functional SBE. Here we first asked if this peculiarity of the LAMA3 promoter may also somehow be involved in Smad4-dependent responses to TNFα.

**Figure 6:**
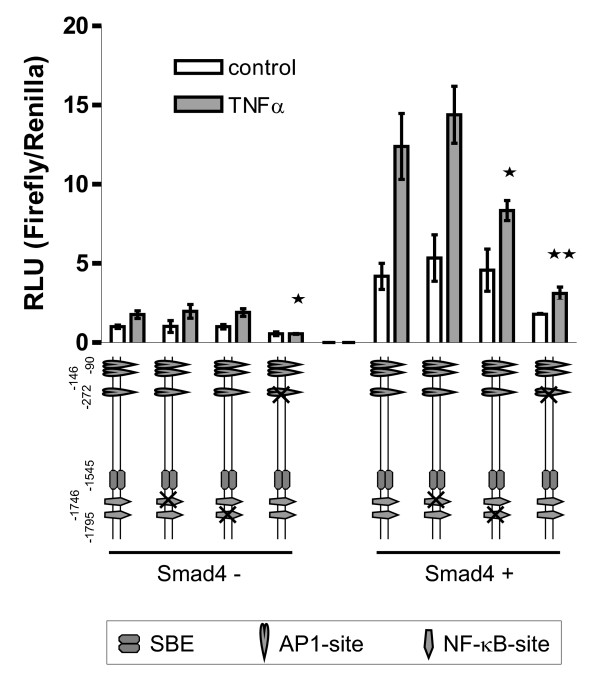
**Analysis of regulatory sites involved in TNFα responses of the LAMA3 promoter by transient transfection of promoter-reporter constructs**. Normalized promoter activities of LAMA3 wild-type and mutated promoter constructs. SW480 cells were plated in 96-well plates and transfected with the indicated promoter constructs using the Dual-Luciferase-Reporter Assay System (Promega). An NF-κB site is involved in promoter responses in Smad4-positive but not in Smad4-negative cells. An AP1 site is involved in TNFα responsiveness in a Smad4-independent manner. Bars show the mean value of three approaches with the standard error of the mean. Statistical analysis was carried out by t test (one-tailed, *GraphPad Prism 4.00*). *P < 0.05, **P < 0.01

A luciferase construct harbouring the 2 kb region upstream promoter region from the transcription start site reflected TNFα responses of the endogenous LAMA3 gene (Figure 6). Responses to a corresponding construct with the SBE site at position -1.5 kb mutated were indistinguishable (data not shown). *In silico *analysis revealed that this promoter region harbors two NF-κB sites located at positions -1.75 and -1.80 kb. Mutation of the upstream NF-κB site strongly reduced TNFα-inducibility of LAMA3 in Smad4-positive cells but did not affect LAMA3 responses in Smad4-negative cells. Mutation of the downstream site was without effect. AP1 sites have previously been shown to be involved in basal and TGFβ-induced LAMA3 expression [19]. Mutation of the most upstream AP1 site at position -272 also led to reduced TNFα inducibility of LAMA3 expression in Smad4-positive cells and completely abolished the low-level TNFα inducibility of LAMA3 expression in Smad4-negative cells. In summary, we here have implicated an NF-κB site and to a lesser extent an AP1 site in Smad4-dependent TNFα induction of LAMA3 expression.

In contrast, mutation of a (cryptic) NF-κB binding site at position -166, the only NF-κB binding site within the 0.8 kb promoter fragment of LAMC2, did not reduce TNFα inducibility (data not shown). AP1 sites previously implicated in TGFβ induction of LAMB3 and LAMC2 are also involved in TNFα induction of these genes in Smad4-positive and in Smad4-negative cells (Additional file 4).

## Discussion

Invading tumor cells are maximally exposed to growth factors and cytokines expressed by stromal cell types. Among these, macrophages have previously been shown to induce angiogenesis [23] and to enhance invasion through the secretion of TNFα [24]. TNFα in cooperation with TGFβ dramatically enhanced EMT [24]. On the other hand normal intestinal epithelial cells respond to TNFα and TGFβ with an increase in the expression of heterotrimeric laminin-332 [25]. We therefore focused on the analysis of laminin-332 expression in response to cytokines TGFβ and TNFα in cell models adequate to reflect the molecular progression of colorectal cancer in vitro. To this end we used pairs of cell clones derived from the human Smad4-deficient colorectal cancer cell lines SW480 and SW620, in which Smad4 expression was stably restored. Responses of Smad4-reexpressing cancer cells were compared to responses of LT97 cells, a cell line derived from a late adenoma and carrying inactivated APC as well as an activated Ki-ras oncogene [20]. LT97 cells secreted increased amounts of heterotrimeric laminin-332 in response to TGFβ and TNFα, respectively, and showed extensive synergistic induction of laminin-332 in response to the combination of both cytokines. Smad4-reexpressing colon cancer cells displayed similar, although less pronounced effects. These results are consistent with observations in vivo, that colorectal adenoma cells in the vicinity of infiltrating inflammatory cells display thickening of the basement membrane with streak-like deposits of laminin-332 [10]. Likewise, intestinal epithelial cells in patients afflicted with Morbus Crohn show increased levels of TNFα and induced expression of the constituents of laminin-332 [26,27]. The response of (normal and) premalignant cells to increase expression of laminin-332 may thus be interpreted as a defense mechanism against an inflammatory attack by strengthening the basement membrane barrier.

Synergistic induction of laminin-332 in response to TGFβ and TNFα was Smad4-dependent as it did not occur in Smad4-deficient SW480 and SW620 cells. Smad4-deficient cells can induce the expression of the (β3 and) γ2 chain of laminin-332 in response to TNFα whereas TNFα induction of the α3 chain is Smad4-dependent. These results indicate that loss of Smad4 may represent a genetic alteration in the carcinogenic process that can lead to uncoupled regulation of the three genes encoding laminin-332 in response to inflammatory cytokines. We have shown previously that the molecular mechanisms of Smad4-dependent regulation of the three promoters encoding laminin-332 are surprisingly complex. Concerning basal and TGFβ-induced expression levels Smad4 is essential for positive regulation of all three genes. The molecular mechanisms underlying this regulation, however, are significantly divergent between the LAMA3 promoter as compared to the LAMB3 and LAMC2 promoters [19]. Here we show that Smad4 is essential for TNFα induction of LAMA3 but not of LAMB3 and LAMC2 and that AP1 and NF-κB sites are involved in TNFα-mediated Smad4-dependent LAMA3 induction. Unraveling transcription factor complexes built in response to cytokines and active at the three promoters will require further detailed analyses.

We here focus on functional consequences of uncoupled regulation of the three laminin-332 chains in response to TNFα. The prevailing view suggests that the β3 and γ2 chains first form a heterodimer intracellularly, which then binds to α3 followed by rapid secretion of the heterotrimer [28]. Uncoupled induction at the mRNA level in response to TNFα therefore let us expect an intracellular accumulation of the γ2 chain in Smad4-deficient cells. Despite repeated attempts, however, intracellular γ2 could not be detected by Western blotting of cell homogenates. Rather, Smad4-deficient cells release the γ2 chain in a monomeric form and in two complexes with as yet unidentified proteins as shown by Western blotting and unequivocally confirmed by mass spectrometry.

What are the functional implications of the release of monomeric γ2? We have shown here, that increased amounts of γ2 are associated with increased migration and assume that secreted γ2 may somehow promote tumor invasion. The release of γ2 may impinge on the composition of the extracellular matrix, alter its functional characteristics and so indirectly affect cell adhesion and migration. Interaction of γ2 with various ECM molecules including collagen, perlecan and fibulin has been reported [29,30]. Alternatively, monomeric γ2 may directly affect the tumor cells by interacting with cellular receptors followed by effects on cell signaling which subsequently may result in cell migration. The predominant laminin-receptors are the integrins. Whereas interaction of laminin-332 with cells is predominantly mediated via integrins α3β1 or α6β4 through binding to the laminin-like globular domains of the α3 laminin chain, the γ2 chain can bind to α2β1 integrin [30]. Interestingly, it is known that domain III of the γ2 chain can also directly interact with the EGF-receptor [31]. EGF signaling is a major stimulus for cell migration.

Clues to the functional relevance of γ2 secreted from cells may come from further analysis of γ2 complexes in conditioned media. Western blotting as well as results from mass spectrometry indicated that similar amounts of γ2 are present under non-reducing conditions at 140 kDa corresponding to the monomeric form and at 240 kDa corresponding to a γ2 protein complex; thus, we assume that the laminin-γ2 chain may interact with another protein of approximately 100 kDa in size. The proteomic analysis surprisingly provided evidence for another as yet unknown γ2 complex of about 400 kDa in size. As soon as alternative γ2 binding partners will be identified their functional relevance for tumor cell migration and invasion can be addressed.

In summary, our results provide evidence for a sequence of events, in which loss of the Smad4 leads to induction of the laminin γ2 chain in response to TNFα followed by the release of monomeric laminin γ2 which exerts a proinvasive effect. In conclusion, we here suggest a novel mechanism that may underlie the switch to invasive tumor growth upon loss of the tumor suppressor Smad4.

A large variety of growth factors and cytokines can be expressed at the invasive margin of carcinomas; some of them have previously been suspected to underlie relative overexpression of γ2. For example, Olsen at al. investigated the involvement of HGF and found synergistic induction of γ2 but not α3 by HGF and TGFβ [32]. LAMC2 is an established β-catenin target gene and nuclear β-catenin has been reported to correlate with intracellular accumulation of γ2 at invasive margins and in budding tumor cells [15]. Thus, upstream ligands of the wnt gene family induced upon cancer progression may also represent putative inducers of overexpressed γ2. Interestingly, the expression of wnts 2 and 5 has specifically been found in macrophages associated with colon tumors [33]. Activated macrophages can indirectly promote Wnt signaling through TNFα [34,35]. We here present data showing that tumor cell responses to TNFα and to the combination of TNFα and TGFβ critically depend on Smad4. As extensive crosstalk mechanisms exist between Wnt/β-catenin and TGFβ/Smad pathways [36-38] the detailed understanding of laminin regulation will require future investigations based on an integrated view of signaling networks in normal and oncogenically programmed cells and their respective responses to a dynamic cytokine milieu.

## Conclusions

The laminin-γ2 chain, which is physiologically deposited in basement membranes as a component of the heterotrimeric laminin-332, is an impressive marker of invasive margins of aggressive carcinomas. In the present study we show that loss of the tumor suppressor Smad4 may be one of the molecular mechanisms that can lead to this relative overexpression of the laminin-γ2 chain in response to the inflammatory cytokine TNFα. Moreover, we show that this uncoupled expression leads to the release of laminin-γ2 which in turn promotes tumor cell migration. We have thus unraveled a novel molecular mechanism of how loss of the tumor suppressor Smad4 may promote the carcinogenic process in vivo, where tumor cells interact with stromal cell types and respond to inflammatory cytokines like TNFα expressed by macrophages at the tumor host interface.

## Methods

### Cell culture and conditioned media

The human colorectal carcinoma cell lines SW480 and SW620 were obtained from the American Type Culture Collection, the human colon adenoma cell line LT97-2 was kindly provided by M Marian (Vienna, Austria). LT97 cells were maintained in Ham's F12 medium with supplements as described [20]. All other cells were maintained in Dulbecco's Modified Eagle Medium (DMEM) supplemented with 10% fetal calf serum, 2 mM glutamine, 100 U/mL penicillin and 100 μg/mL streptomycin. When indicated, cells were incubated with 5 ng/mL of TGFβ_1 _(R&D systems, Minneapolis, MN, USA) and 30 ng/mL of TNFα (Pan Biotech, Aidenbach, Germany) in serum reduced medium (0.5% FCS). Preparation of proteins from serum-free conditioned media was performed as described previously [39].

### Production of polyclonal antibody 2140

GST-tagged recombinant laminin-γ2 was produced by expressing pGEX γ2lam5 (kindly provided by Dr. M. Failla, IDI-IRCCS, Roma, Italy) in E.coli. Affinity-purified laminin γ2-GST protein was confirmed by mass spectrometry (LSMDO, CNRS-EPCM, UMR7509, Strasbourg, France) and injected into rabbits. Antibodies were verified by immunoblotting on HT29-MTX cells and by immunofluorescence on human intestines (not shown) giving identical but stronger signals than MAB19562 (Chemicon, Hampshire, UK).

### Western blotting and RNA analyses

For laminin Western blots, samples were subjected to SDS-PAGE on either 8% polyacrylamide gels or on NuPAGE Novex 3-8% Tris-Acetate gels (Invitrogen, Karlsruhe, Germany) and run under conditions either with (reducing conditions to analyze single chain expression) or without dithiothreitol (non-reducing reducing conditions). Heterotrimeric laminin-332, dimeric and monomeric laminin-γ2 under non-reducing conditions were detected with monoclonal antibody MAB19562 (Chemicon) and polyclonal antibody 2140. The blots were incubated with a secondary antibody directly coupled with a fluorescent dye (Alexa Fluor 680; Alexa Fluor 800; Invitrogen and Rockland). Signals were detected using the Odyssey Infrared Imaging System (LI-COR Biosciences, Bad Homburg, Germany) which allows for a digital quantification of signals over a wide linear range of signal intensities. RNA analyses were performed according to standard procedures by Northern blot hybridization and by qRT-PCR as described previously [16,19,21].

### Mass spectrometry and mass spectrometric data analysis

Mass spectrometry and mass spectrometric analysis are described in detail in the additional information. In brief, tryptic digest were analyzed by nano-HPLC/ESI-MS/MS using the UltiMate™ 3000 HPLC system (Dionex LC Packings, Idstein, Germany) online coupled to an LTQ Orbitrap XL instrument (Thermo Fisher Scientific, Bremen, Germany). Reversed-phase (RP) capillary HPLC separations were performed as described previously [40].

Peak lists of MS/MS spectra were imported into ProteinScape (version 1.3, Bruker Daltonics, Bremen, Germany) and subsequently correlated with the human International Protein Index database (human IPI v3.41, http://www.ebi.ac.uk) containing 72155 protein entries using MASCOT (release version 2.2) [41]. To enable the estimation of a false discovery rate (FDR), the database was concatenated with a duplicate of itself in which the amino acid sequence of each protein entry was randomly shuffled [42]. Protein hits up to an accumulated FDR of 5% were considered as true positive identifications.

### Migration assay and transient LAMA3 knockdown

Cells in 500 μL media were added to the upper compartment of 12 well plates supplemented with inserts (8 μm pore size; BD Falcon). Cytokines were added one day later and cells incubated for another 72 hours at 37°C. Cells which had passed the pore membrane were quantified using Cell Titer Glo (Promega, Madison, WI, USA) in accordance with the manufacturer's recommendations.

For siRNA experiments cells were grown to a confluency of 50% and transfected with ON-TARGETplus siRNA (LAMA3, J-011071-05, Dharmacon, Lafayette, CO, USA) or Dharmacon ON-TARGETplus Nontargeting siRNA as a control, respectively, using Dharmafect (Dharmacon). For migration assays cells were plated into transwells 24 h after transfection.

### Promoter analyses

Promoter construction and transient transfections were performed as previously described [16,19] with minor modifications. Cells were grown to a confluency of approximately 50-70% in 96-well plates, medium was changed to serum reduced medium (0.5% FCS) and cells were transfected using Effectene (Qiagen, Hilden, Germany). The next day cytokines were added and cells were harvested 4 h and 24 h after transfection. Luciferase assays were carried out as triplicates and quantified using a luminometer (GloMax™ 96 Microplate, Promgea, Madison, WI, USA) and the Dual-Luciferase-Reporter Assay System (Promega).

## Competing interests

The authors declare that they have no competing interests.

## Authors' contributions

DZ designed the study, carried out Western Blot, RNA and promoter analyses, performed transient knockdown and migration experiments and drafted the manuscript. BW performed mass spectrometry analyses. MBM and HB participated in expression analyses. SK-S, HEM and WS contributed to the design of the study. PS-A established the novel polyclonal laminin-γ2 antibody 2140 and contributed to the manuscript. IS-W is the PI, designed the study and drafted the manuscript. All authors have read and approved the final manuscript.

## Supplementary Material

Additional file 1 **Restoration of TGFβ responsiveness through re-expression of Smad4**. Smad4 expression was stably restored by retroviral transduction in Smad4-deficient human SW620 colon carcinoma cells. **(A) **Western blot analysis for the human Smad4 protein on total protein extracts of each three Smad4-negative and six Smad4-positive clones of SW620 cells (TJ: empty vector control clones, DTJ: Smad4- (DPC4) positive clones). **(B and C) **Transient transfections with p3Tplux (B) and p6SBE (C) reporter vectors of each four Smad4-negative and five Smad4-positive derivates of SW620 cells. Normalized promoter activity of p3Tplux (a fusion construct of the PAI-1 and collagenase-1 promoters harboring AP1 sites) and p6SBE (a 6fold concatemer of the SBE) as analyzed in transient transfections of TGFβ-treated (24 h) and -untreated Smad4 negative and Smad4 re-expressing cells. Transient transfection experiments were repeated in triplicates. The bars show the mean values with the standard error of the mean. For further experiments we defined a standard clone set consisting of clones TJ3, 9 and 10 and DTJ8, 16 and 21. Click here for file

Additional file 2 **Synergistic effect of TGFβ and TNFα on the expression of LAMA3, LAMB3 and LAMC2 genes by Smad4-reconstituted human colorectal cancer cells and uncoupled responses of Smad4-deficient cells**. **(A and B) **Semi-quantitative RT-PCR analysis of the LAMA3, LAMB3 and LAMC2 genes prepared with RNAs from SW480 cells treated with recombinant TGFβ and TNFα for 4 h (A) and 24 h (B). Shown in each bar is the mean +/- standard error of 10 measurements. **(C) **Northern blot analysis of the LAMA3, LAMB3 and LAMC2 genes prepared with RNAs from SW620 cells treated with recombinant TGFβ and TNFα for 24 h. Signals were quantified by phosphorimage analysis and normalized for GAPDH expression (n = 3). Click here for file

Additional file 3 **Mass spectrometry of proteins and protein complexes reactive with laminin-γ2 specific antibodies as indicated in Figure 4a**. Proteins were identified through SDS-PAGE combined with nano-high performance liquid chromatography coupled online with electrospray ionization tandem mass spectrometry. MS/MS data were used for protein identification by performing searches in the human IPI database with Mascot and for the calculation of spectral counts as a relative quantitative measure for protein abundance. Proteins were identified with a false discovery rate of 5%. Click here for file

Additional file 4 **TNFα induction of LAMB3 and LAMC2 is conferred through AP1 binding sites**. Normalized promoter activities of LAMB3 **(A) **and LAMC2 **(B) **wild-type and mutated promoter constructs. SW480 cells were plated in 96-well plates and transfected with the indicated promoter constructs using the Dual-Luciferase-Reporter Assay System (Promega). Mutagenesis of both AP1 sites in the LAMB3 promoter significantly reduced TNFα responsiveness in Smad4 reexpressing cells. TNFα induction of LAMC2 is conferred through the upstream AP1 site in a Smad4-independent manner. Bars show the mean value of three experiments with the standard error of the mean. Click here for file

## References

[B1] Simon-Assmann P Bolcato-Bellemin AL Turck N Piccinni S Olsen J Launay JF Lefebvre O Kedinger M Johnstone P Basement membrane laminins in normal and pathological intestine Disease Progression and Carcinogenesis in the Gastrointestinal Tract 2003 London, Kluwer Academic Publishers 223 239

[B2] Teller IC Beaulieu JF Interactions between laminin and epithelial cells in intestinal health and disease Expert Rev Mol Med 2001 9 1 18 14585148 10.1017/S1462399401003623

[B3] Kalluri R Basement membranes: structure, assembly and role in tumour angiogenesis Nat Rev Cancer 2003 9 6 422 433 10.1038/nrc1094 12778132

[B4] Givant-Horwitz V Davidson B Reich R Laminin-induced signaling in tumor cells Cancer Lett 2005 9 1 1 10 10.1016/j.canlet.2004.08.030 15890231

[B5] Tzu J Marinkovich MP Bridging structure with function: structural, regulatory, and developmental role of laminins Int J Biochem Cell Biol 2008 9 2 199 214 10.1016/j.biocel.2007.07.015 17855154 PMC2192629

[B6] Lu W Miyazaki K Mizushima H Nemoto N Immunohistochemical distribution of laminin-5 gamma2 chain and its developmental change in human embryonic and foetal tissues Histochem J 2001 9 11-12 629 637 10.1023/A:1016350316926 12197671

[B7] Simon-Assmann P Lefebvre O Bellissent-Waydelich A Olsen J Orian-Rousseau V De Arcangelis A The laminins: role in intestinal morphogenesis and differentiation Ann N Y Acad Sci 1998 9 46 64 10.1111/j.1749-6632.1998.tb11110.x 9928369

[B8] Teller IC Auclair J Herring E Gauthier R Menard D Beaulieu JF Laminins in the developing and adult human small intestine: relation with the functional absorptive unit Dev Dyn 2007 9 7 1980 1990 10.1002/dvdy.21186 17503455

[B9] Lohi J Laminin-5 in the progression of carcinomas Int J Cancer 2001 9 6 763 767 10.1002/ijc.1539 11745475

[B10] Sordat I Bosman FT Dorta G Rousselle P Aberdam D Blum AL Sordat B Differential expression of laminin-5 subunits and integrin receptors in human colorectal neoplasia J Pathol 1998 9 1 44 52 10.1002/(SICI)1096-9896(199805)185:1<44::AID-PATH46>3.0.CO;2-A 9713359

[B11] Spaderna S Schmalhofer O Hlubek F Berx G Eger A Merkel S Jung A Kirchner T Brabletz T A transient, EMT-linked loss of basement membranes indicates metastasis and poor survival in colorectal cancer Gastroenterology 2006 9 3 830 840 10.1053/j.gastro.2006.06.016 16952552

[B12] Katayama M Sekiguchi K Laminin-5 in epithelial tumour invasion J Mol Histol 2004 9 3 277 286 10.1023/B:HIJO.0000032359.35698.fe 15339047

[B13] Giannelli G Antonaci S Biological and clinical relevance of Laminin-5 in cancer Clin Exp Metastasis 2000 9 6 439 443 10.1023/A:1011879900554 11592300

[B14] Miyazaki K Laminin-5 (laminin-332): Unique biological activity and role in tumor growth and invasion Cancer Sci 2006 9 2 91 98 10.1111/j.1349-7006.2006.00150.x 16441418 PMC11159065

[B15] Hlubek F Jung A Kotzor N Kirchner T Brabletz T Expression of the invasion factor laminin gamma2 in colorectal carcinomas is regulated by beta-catenin Cancer Res 2001 9 22 8089 8093 11719433

[B16] Zapatka M Zboralski D Radacz Y Bockmann M Arnold C Schoneck A Hoppe S Tannapfel A Schmiegel W Simon-Assmann P Schwarte-Waldhoff I Basement membrane component laminin-5 is a target of the tumor suppressor Smad4 Oncogene 2007 9 10 1417 1427 10.1038/sj.onc.1209918 16953227

[B17] Massague J Seoane J Wotton D Smad transcription factors Genes Dev 2005 9 23 2783 2810 10.1101/gad.1350705 16322555

[B18] Schmierer B Hill CS TGFbeta-SMAD signal transduction: molecular specificity and functional flexibility Nat Rev Mol Cell Biol 2007 9 12 970 982 10.1038/nrm2297 18000526

[B19] Zboralski D Bockmann M Zapatka M Hoppe S Schoneck A Hahn SA Schmiegel W Schwarte-Waldhoff I Divergent mechanisms underlie Smad4-mediated positive regulation of the three genes encoding the basement membrane component laminin-332 (laminin-5) BMC Cancer 2008 9 215 10.1186/1471-2407-8-215 18664273 PMC2525660

[B20] Richter M Jurek D Wrba F Kaserer K Wurzer G Karner-Hanusch J Marian B Cells obtained from colorectal microadenomas mirror early premalignant growth patterns in vitro Eur J Cancer 2002 9 14 1937 1945 10.1016/S0959-8049(02)00158-2 12204677

[B21] Schwarte-Waldhoff I Klein S Blass-Kampmann S Hintelmann A Eilert C Dreschers S Kalthoff H Hahn SA Schmiegel W DPC4/SMAD4 mediated tumor suppression of colon carcinoma cells is associated with reduced urokinase expression Oncogene 1999 9 3152 3158 10.1038/sj.onc.1202641 10340387

[B22] Liu H Sadygov RG Yates RG III A model for random sampling and estimation of relative protein abundance in shotgun proteomics Anal Chem 2004 9 14 4193 4201 10.1021/ac0498563 15253663

[B23] Etoh T Shibuta K Barnard GF Kitano S Mori M Angiogenin expression in human colorectal cancer: the role of focal macrophage infiltration Clin Cancer Res 2000 9 9 3545 3551 10999742

[B24] Bates RC Mercurio AM Tumor necrosis factor-alpha stimulates the epithelial-to-mesenchymal transition of human colonic organoids Mol Biol Cell 2003 9 5 1790 1800 10.1091/mbc.E02-09-0583 12802055 PMC165077

[B25] Francoeur C Escaffit F Vachon PH Beaulieu JF Proinflammatory cytokines TNF-alpha and IFN-gamma alter laminin expression under an apoptosis-independent mechanism in human intestinal epithelial cells Am J Physiol Gastrointest Liver Physiol 2004 9 3 G592 598 10.1152/ajpgi.00535.2003 15087281

[B26] Bouatrouss Y Herring-Gillam FE Gosselin J Poisson J Beaulieu JF Altered expression of laminins in Crohn's disease small intestinal mucosa Am J Pathol 2000 9 1 45 50 10623652 10.1016/S0002-9440(10)64704-9 PMC1868644

[B27] Reimund JM Wittersheim C Dumont S Muller CD Kenney JS Baumann R Poindron P Duclos B Increased production of tumour necrosis factor-alpha interleukin-1 beta, and interleukin-6 by morphologically normal intestinal biopsies from patients with Crohn's disease Gut 1996 9 5 684 689 10.1136/gut.39.5.684 9026483 PMC1383392

[B28] Matsui C Wang CK Nelson CF Bauer EA Hoeffler WK The assembly of laminin-5 subunits J Biol Chem 1995 9 40 23496 23503 10.1074/jbc.270.40.23496 7559513

[B29] Sasaki T Gohring W Mann K Brakebusch C Yamada Y Fassler R Timpl R Short arm region of laminin-5 gamma2 chain: structure, mechanism of processing and binding to heparin and proteins J Mol Biol 2001 9 4 751 763 10.1006/jmbi.2001.5176 11733994

[B30] Tsuruta D Kobayashi H Imanishi H Sugawara K Ishii M Jones JC Laminin-332-integrin interaction: a target for cancer therapy? Curr Med Chem 2008 9 20 1968 1975 10.2174/092986708785132834 18691052 PMC2992754

[B31] Schenk S Hintermann E Bilban M Koshikawa N Hojilla C Khokha R Quaranta V Binding to EGF receptor of a laminin-5 EGF-like fragment liberated during MMP-dependent mammary gland involution J Cell Biol 2003 9 1 197 209 10.1083/jcb.200208145 12695504 PMC2172889

[B32] Olsen J Kirkeby LT Brorsson MM Dabelsteen S Troelsen JT Bordoy R Fenger K Larsson LI Simon-Assmann P Converging signals synergistically activate the LAMC2 promoter and lead to accumulation of the laminin gamma 2 chain in human colon carcinoma cells Biochem J 2003 9 Pt 1 211 221 10.1042/BJ20021454 12519076 PMC1223269

[B33] Smith K Bui TD Poulsom R Kaklamanis L Williams G Harris AL Up-regulation of macrophage wnt gene expression in adenoma-carcinoma progression of human colorectal cancer Br J Cancer 1999 9 3 496 502 10.1038/sj.bjc.6690721 10507776 PMC2362915

[B34] DeNardo DG Johansson M Coussens LM Inflaming gastrointestinal oncogenic programming Cancer Cell 2008 9 1 7 9 10.1016/j.ccr.2008.06.010 18598939

[B35] Oguma K Oshima H Aoki M Uchio R Naka K Nakamura S Hirao A Saya H Taketo MM Oshima M Activated macrophages promote Wnt signalling through tumour necrosis factor-alpha in gastric tumour cells Embo J 2008 9 12 1671 1681 10.1038/emboj.2008.105 18511911 PMC2413189

[B36] Labbe E Letamendia A Attisano L Association of Smads with lymphoid enhancer binding factor 1/T cell-specific factor mediates cooperative signaling by the transforming growth factor-beta and wnt pathways Proc Natl Acad Sci USA 2000 9 15 8358 8363 10.1073/pnas.150152697 10890911 PMC26952

[B37] Letamendia A Labbe E Attisano L Transcriptional regulation by Smads: crosstalk between the TGF-beta and Wnt pathways J Bone Joint Surg Am 2001 9 Suppl 1(Pt 1) S31 39 11263663

[B38] Nishita M Hashimoto MK Ogata S Laurent MN Ueno N Shibuya H Cho KW Interaction between Wnt and TGF-beta signalling pathways during formation of Spemann's organizer Nature 2000 9 6771 781 785 10.1038/35001602 10693808

[B39] Volmer MW Radacz Y Hahn SA Klein-Scory S Stuhler K Zapatka M Schmiegel W Meyer HE Schwarte-Waldhoff I Tumor suppressor Smad4 mediates downregulation of the anti-adhesive invasion-promoting matricellular protein SPARC: Landscaping activity of Smad4 as revealed by a "secretome" analysis Proteomics 2004 9 5 1324 1334 10.1002/pmic.200300703 15188399

[B40] Schaefer H Chervet JP Bunse C Joppich C Meyer HE Marcus K A peptide preconcentration approach for nano-high-performance liquid chromatography to diminish memory effects Proteomics 2004 9 9 2541 2544 10.1002/pmic.200300801 15352228

[B41] Perkins DN Pappin DJ Creasy DM Cottrell JS Probability-based protein identification by searching sequence databases using mass spectrometry data Electrophoresis 1999 9 18 3551 3567 10.1002/(SICI)1522-2683(19991201)20:18<3551::AID-ELPS3551>3.0.CO;2-2 10612281

[B42] Stephan C Reidegeld KA Hamacher M van Hall A Marcus K Taylor C Jones P Muller M Apweiler R Martens L Körting G Chamrad DC Thiele H Blüggel M Parkinson D Binz PA Lyall A Meyer HE Automated reprocessing pipeline for searching heterogeneous mass spectrometric data of the HUPO Brain Proteome Project pilot phase Proteomics 2006 9 18 5015 5029 10.1002/pmic.200600294 16927432

