# Supplementary figures and images for: Uncoupled responses of Smad4-deficient cancer cells to TNFα result in secretion of monomeric laminin-γ2
Source: Mol Cancer. 2010 Mar 22;9:65. doi: 10.1186/1476-4598-9-65 (PMC2853515; doi:10.1186/1476-4598-9-65)

**A**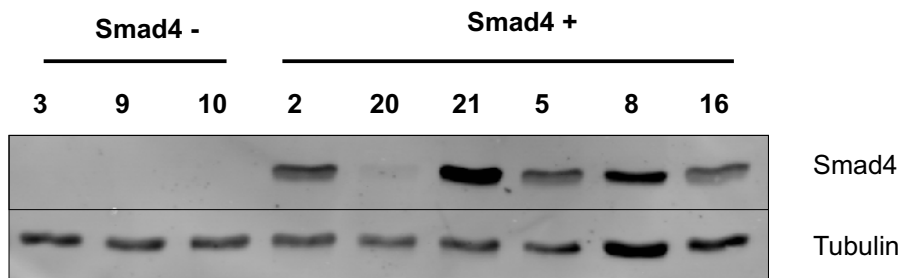**B**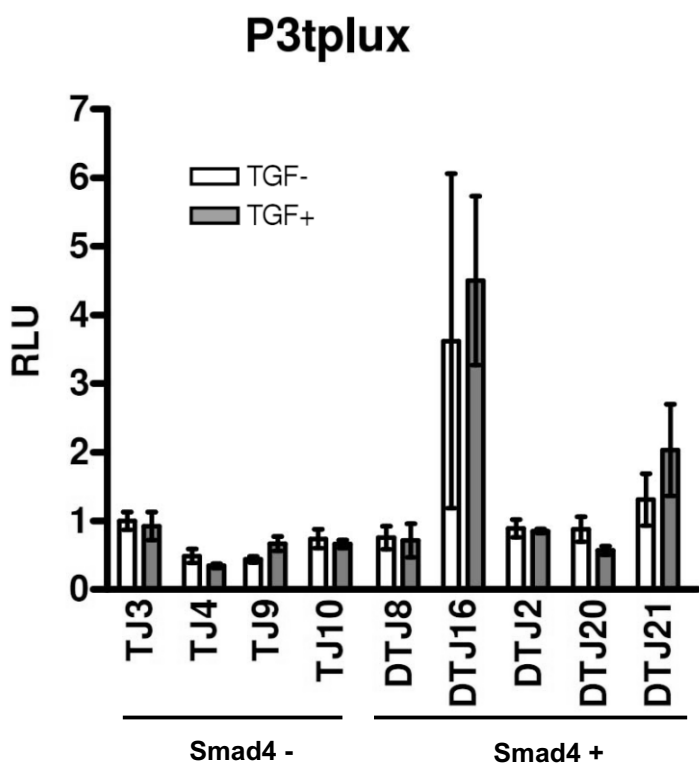**C**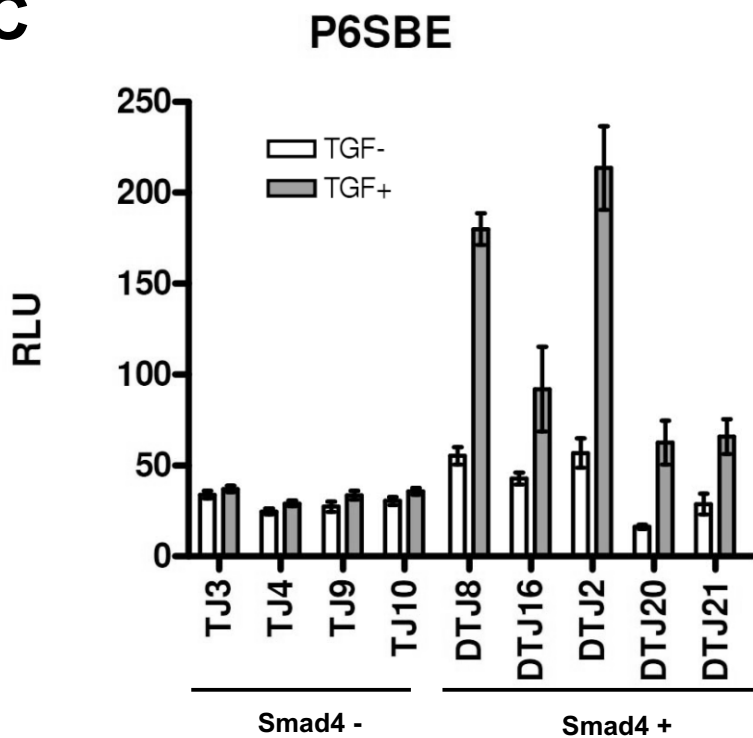

Supplement: Additional file 1 — Restoration of TGFβ responsiveness through re-expression of Smad4. Smad4 expression was stably restored by retroviral transduction in Smad4-deficient human SW620 colon carcinoma cells. (A) Western blot analysis for the human Smad4 protein on total protein extracts of each three Smad4-negative and six Smad4-positive clones of SW620 cells (TJ: empty vector control clones, DTJ: Smad4- (DPC4) positive clones). (B and C) Transient transfections with p3Tplux (B) and p6SBE (C) reporter vectors of each four Smad4-negative and five Smad4-positive derivates of SW620 cells. Normalized promoter activity of p3Tplux (a fusion construct of the PAI-1 and collagenase-1 promoters harboring AP1 sites) and p6SBE (a 6fold concatemer of the SBE) as analyzed in transient transfections of TGFβ-treated (24 h) and -untreated Smad4 negative and Smad4 re-expressing cells. Transient transfection experiments were repeated in triplicates. The bars show the mean values with the standard error of the mean. For further experiments we defined a standard clone set consisting of clones TJ3, 9 and 10 and DTJ8, 16 and 21. [file 1476-4598-9-65-S1.PDF]

**A****4h**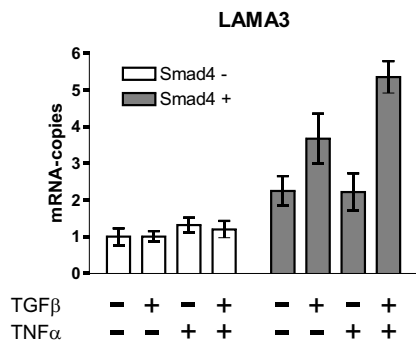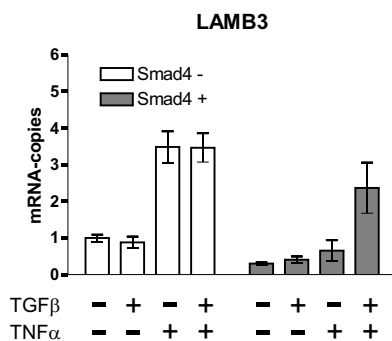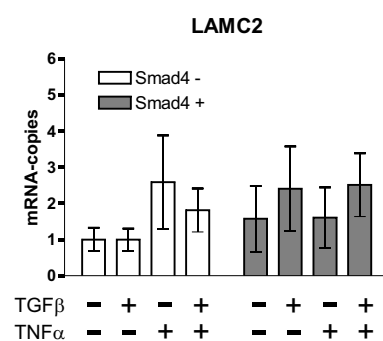**B****24h**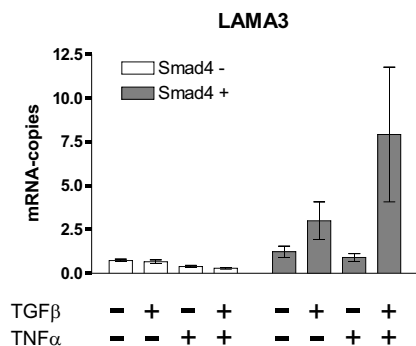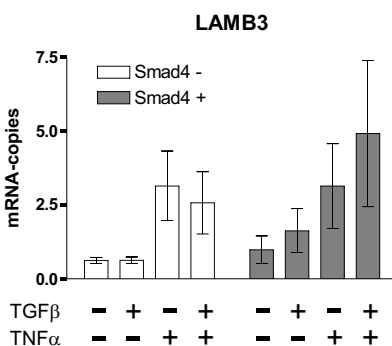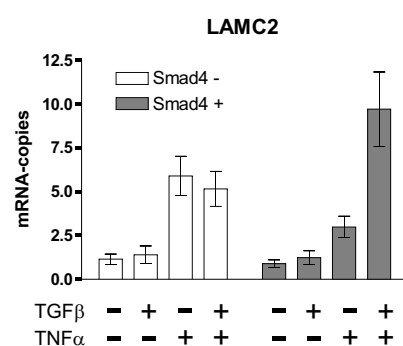**C****24h**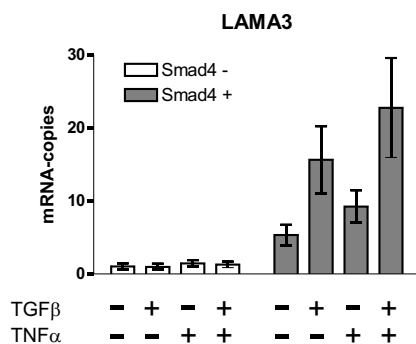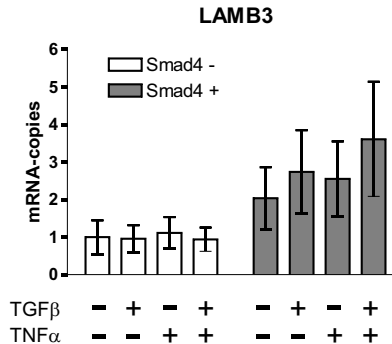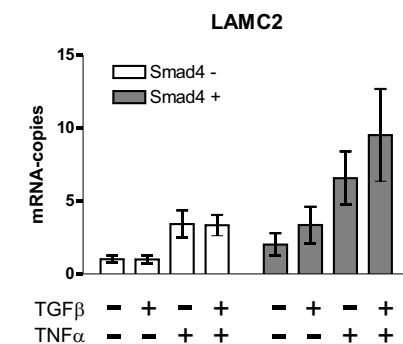

Supplement: Additional file 2 — Synergistic effect of TGFβ and TNFα on the expression of LAMA3, LAMB3 and LAMC2 genes by Smad4-reconstituted human colorectal cancer cells and uncoupled responses of Smad4-deficient cells. (A and B) Semi-quantitative RT-PCR analysis of the LAMA3, LAMB3 and LAMC2 genes prepared with RNAs from SW480 cells treated with recombinant TGFβ and TNFα for 4 h (A) and 24 h (B). Shown in each bar is the mean +/- standard error of 10 measurements. (C) Northern blot analysis of the LAMA3, LAMB3 and LAMC2 genes prepared with RNAs from SW620 cells treated with recombinant TGFβ and TNFα for 24 h. Signals were quantified by phosphorimage analysis and normalized for GAPDH expression (n = 3). [file 1476-4598-9-65-S2.PDF]

**A**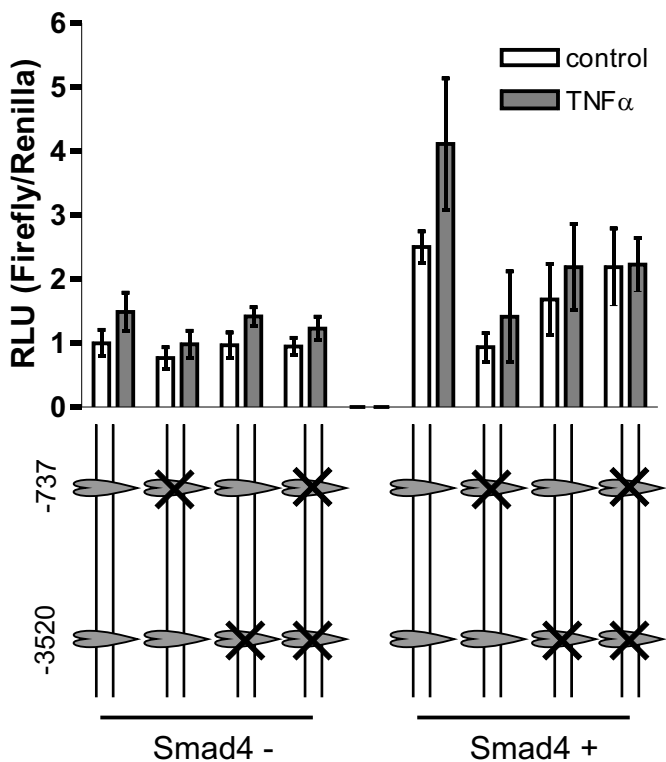**B**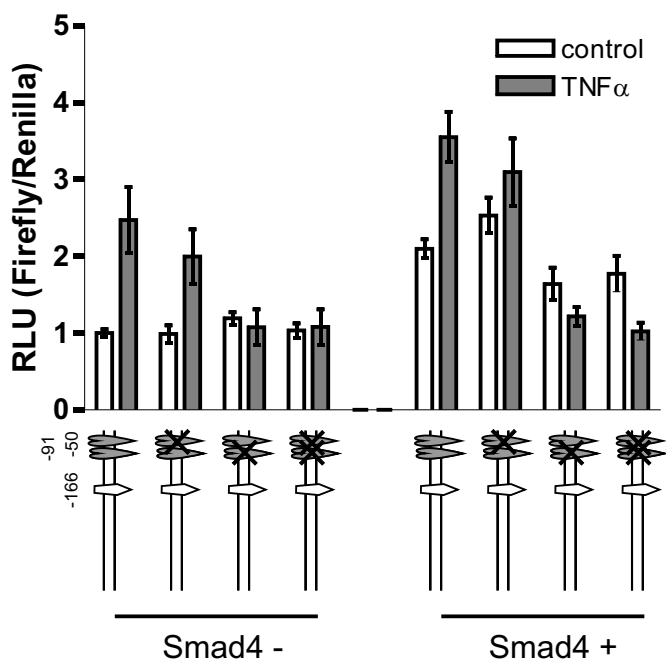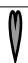

AP1-site

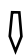cryptic NF- $\kappa$ B-site

Supplement: Additional file 4 — TNFα induction of LAMB3 and LAMC2 is conferred through AP1 binding sites. Normalized promoter activities of LAMB3 (A) and LAMC2 (B) wild-type and mutated promoter constructs. SW480 cells were plated in 96-well plates and transfected with the indicated promoter constructs using the Dual-Luciferase-Reporter Assay System (Promega). Mutagenesis of both AP1 sites in the LAMB3 promoter significantly reduced TNFα responsiveness in Smad4 reexpressing cells. TNFα induction of LAMC2 is conferred through the upstream AP1 site in a Smad4-independent manner. Bars show the mean value of three experiments with the standard error of the mean. [file 1476-4598-9-65-S4.PDF]
